# What would my avatar do? Gaming, pathology, and risky decision making

**DOI:** 10.3389/fpsyg.2013.00609

**Published:** 2013-09-10

**Authors:** Kira Bailey, Robert West, Judson Kuffel

**Affiliations:** ^1^Social Cognitive Neuroscience Laboratory, Department of Psychological Sciences, University of MissouriColumbia, MO, USA; ^2^Temporal Dynamics of Attention & Memory Laboratory, Department of Psychology, Iowa State UniversityAmes, IA, USA; ^3^Educational Psychology & Foundations, University of Northern IowaCedar Falls, IA, USA

**Keywords:** video games, decision making, risk, pathological video game use, impulsivity, reward processing

## Abstract

Recent work has revealed a relationship between pathological video game use and increased impulsivity among children and adolescents. A few studies have also demonstrated increased risk-taking outside of the video game environment following game play, but this work has largely focused on one genre of video games (i.e., racing). Motivated by these findings, the aim of the current study was to examine the relationship between pathological and non-pathological video game use, impulsivity, and risky decision making. The current study also investigated the relationship between experience with two of the most popular genres of video games [i.e., first-person shooter (FPS) and strategy] and risky decision making. Consistent with previous work, ~7% of the current sample of college-aged adults met criteria for pathological video game use. The number of hours spent gaming per week was associated with increased impulsivity on a self-report measure and on the temporal discounting (TD) task. This relationship was sensitive to the genre of video game; specifically, experience with FPS games was positively correlated with impulsivity, while experience with strategy games was negatively correlated with impulsivity. Hours per week and pathological symptoms predicted greater risk-taking in the risk task and the Iowa Gambling task, accompanied by worse overall performance, indicating that even when risky choices did not pay off, individuals who spent more time gaming and endorsed more symptoms of pathological gaming continued to make these choices. Based on these data, we suggest that the presence of pathological symptoms and the genre of video game (e.g., FPS, strategy) may be important factors in determining how the amount of game experience relates to impulsivity and risky-decision making.

Past research has demonstrated that video games experience influences cognition and emotion in multiple ways (West and Bailey, 2013). For instance, greater video game experience is associated with decreased use of proactive cognitive control (Kronenberger et al., 2005; Mathews et al., 2005; Bailey et al., 2010), differences in the experience and expression of positive and negative affect (Bartholow et al., 2006; Kirsh and Mounts, 2007; Bailey et al., 2011), and an increase in the number of symptoms associated with ADHD, particularly for individuals who demonstrate pathological video game play (PVP; Gentile, 2009; Gentile et al., 2011; Pawlikowski and Brand, 2011). Findings from numerous studies demonstrate that the efficacy of decision making is moderated by emotion, executive or cognitive control, and the presence of chemical and behavioral addiction (Tanabe et al., 2007; Weber and Johnson, 2009; Figner and Weber, 2011). Given the association between video game experience, PVP, and emotion and cognitive control, one could expect video game experience to have a detrimental effect on the efficacy of decision making. In support of this hypothesis, a few studies have demonstrated that exposure to racing video games can influence real-world decision making related to driving behavior (Fischer et al., 2009; Beullens et al., 2011). The current study extends upon existing evidence by examining the relationship between other genres of video games [i.e., first-person shooter (FPS) and strategy], PVP, and risky decision making in a gambling context.

Research examining the relationship between video games and risky decision making has focused mainly on the effects of racing video games on attitudes toward and engagement in risky driving behaviors (e.g., speeding, fun riding, street racing; see Fischer et al., 2011). Based upon self-report measures, time spent playing racing video games is positively associated with participation in risky driving among adolescents and adults, particularly males (Beullens et al., 2011), and negatively associated with cautious driving (Fischer et al., 2007). Furthermore, laboratory exposure to racing games increases positive attitudes toward risk-taking and greater risk-taking in a computer-simulated driving task (Fischer et al., 2007, 2009), which may be due in part to greater self-perception as a risky driver (Fischer et al., 2009). Additionally, racing video games appear to be most attractive to individuals who are predisposed to an increased risk of automobile accidents and deaths (National Highway Traffic Safety Administration, 2009). Based on these findings, it appears that long and short-term video game exposure can lead to changes in attitudes toward and engagement in behaviors that are exemplified in the game.

Pathological video game use represents a significant problem for 8–9% of children and adolescents (Gentile, 2009; Gentile et al., 2011). Individuals with more PVP symptoms report playing video games more frequently and for longer periods of time, skipping other activities (e.g., homework, chores) to play video games, and using video games to escape their problems more often than their peers. Greater PVP symptomology is also linked to reports of increased aggression and impulsivity, poor performance in school, and elevated levels of symptoms related to depression and ADHD (Gentile et al., 2011).

Pathological gaming may also be related to an increase in risky decision making. Pawlikowski and Brand (2011) examined individual differences in excessive Internet gaming and performance on the Game of Dice task, a measure of risky decision making. In this task, the participant attempts to make as much money as possible by guessing what number would come up from the roll of a 6-sided die. Excessive Internet gamers selected low probability options more frequently than non-gamers, resulting in greater losses. This behavior is similar to that displayed by individuals with gambling problems (Cavedini et al., 2002; Brand et al., 2005). This work suggests that pathological gaming is positively associated with greater impulsivity and risk-taking, above and beyond the amount of time spent gaming.

Evidence from studies examining substance use and problem gambling may provide insight into how PVP influences risky decision making. Substance use (Kirby et al., 1999; Mitchell, 1999; Kim et al., 2011) and problem gambling (Brand et al., 2005; Slutske et al., 2005; Tanabe et al., 2007) are linked to increases in risky decision making through one or more of the following pathways: disrupted executive functions, altered sensitivity to positive and negative outcomes, or increased impulsivity. For example, alcohol dependent patients perform worse on the Iowa Gambling Task (IGT; Kim et al., 2011), taking longer to learn from negative outcomes (i.e., continued selecting cards from the “bad” decks) compared to non-alcohol dependent patients. Pathological gambling has been associated with decreased prefrontal activity in the right hemisphere during the IGT, likely reflecting changes in decision making involving risk (Tanabe et al., 2007). The effects of nicotine on impulsivity have been studied extensively using the temporal discounting (TD) task (e.g., Mitchell, 1999; Ohmura et al., 2005) in which participants choose between smaller rewards delivered immediately or after a short delay and larger rewards delivered after a longer delay (Loewenstein and Thaler, 1989; Read, 2004). Selecting the smaller, immediate reward can be interpreted to reflect greater impulsivity. Cigarette smokers are consistently more impulsive on this task than non-smokers (Mitchell, 1999; Reynolds et al., 2004). Additionally, the extent to which smokers discount delayed monetary gains is correlated with their daily nicotine intake (Reynolds et al., 2004; Ohmura et al., 2005). These findings indicate that substance abuse and problem gambling are positively associated with impulsive selection of immediate rewards, possibly as a result of weakened control over behavior.

Racing video games appear to prime risk-related thoughts and risky driving behaviors; however, it is as yet unclear whether different genres of video games may also prime risky decision making in other domains. There is evidence, however, that certain genres of video games may have differential effects on cognitive control, a set of abilities that allow one to maintain goal-directed information processing (Basak et al., 2008; Bailey et al., 2010). For example, in an individual difference study (Bailey et al., 2010), found that experience with FPS video games was correlated with a reduction in proactive control (active, sustained maintenance of goal-relevant information) and was not correlated with reactive control (just-in-time mobilization of control after conflict is detected; Braver, 2012). Furthermore, Swing (2012) demonstrated that 10 h of FPS experience resulted in a reduction in the use of proactive control in a training study. These findings may indicate that FPS gamers may be more likely to make their decisions in the moment rather than after thoughtful deliberation, a tendency that could manifest as preference for immediate rewards rather than long-term assessment of the risks and benefits. In contrast to FPS games, strategy video games may promote an increase in careful planning and executive control of behavior. Basak et al. (2008) demonstrated that 23.5 h of training on a strategy video game improved task-switching ability and working memory. This area of research is relevant to the current study because similar neural structures are involved in cognitive control and decision making (Steinberg, 2008; Christopoulos et al., 2009). Therefore, the effects of exposure to video games on these brain areas may have consequences for the efficacy of decision making as well.

The goal of the current study was to extend the work of Fischer et al. (2007, 2009) to other video game genres and decision making contexts in order to provide a more comprehensive understanding of how video game experience is related to risky decision making. In order to achieve this goal, multiple decision making tasks involving risk were used. We focused on FPS and strategy video games because of their continued popularity among players (The NDP Group, 2010), as well as their potential to influence decision making in opposing ways. In the current study, individuals reported past video game experience (i.e., hours played per week, PVP symptoms, and genre) and completed a set of questionnaires and computerized tasks assessing risky decision making. Canonical correlation analysis (CCA) was used to examine the latent relationships between video game experience, PVP and sex (i.e., predictor variables), and measures of risky decision making (i.e., dependent variables). Based on previous work (Gentile, 2009; Gentile et al., 2011), we hypothesized that the average number of hours spent playing video games per week and the number of pathological symptoms endorsed would predict increased impulsivity, bias toward immediate or larger rewards, and increased selection of the riskier options. FPS and strategy video games were expected to be differentially associated with risky decision; FPS gamers were expected to be more impulsive and sensitive to rewards, whereas strategy gamers were expected to select fewer risky options and to be more sensitive to negative outcomes. Interactions between hours, PVP, and genre were also examined in order to determine whether the effects of the amount of time spent playing video games and the co-occurrence of pathology would moderate any of the relationships with genre.

## Method

### Participants

Participants were 149 undergraduates (70 females) from Iowa State University ranging in age from 16 to 30 years. Due to an error in the software, data for the testing phase of the probabilistic selection task was lost for one participant. Informed consent was obtained from all participants and they received course credit for their participation. The study was approved by the Institutional Review Board of the university.

### Materials and design

#### Media usage questionnaire

The media usage questionnaire included three higher order questions. Two questions asked the individual to indicate the number of hours spent playing video games on a typical weekday (Question 1, Monday through Friday) or weekend (Question 2, Saturday and Sunday) for each of four time periods (6 am to noon, noon to 6 pm, 6 pm to midnight, and midnight to 6 am). The third question asked the participant to indicate how often s/he plays each of 12 different genres of video games and what video game they spent the most time playing. The dependent variables used were the total number of hours spent playing video games per week and classification as an FPS or strategy video game player (0 or 1) based on the genre of the video game they reported playing most often. The internal reliability was high for the number of hours played (coefficient α = 0.85) and for the amount of experience with genres of video games (coefficient α = 0.87).

#### Pathological gaming scale

A revised version of the PVP scale (Gentile, 2009; Gentile et al., 2011) was composed of 13-items that were based on the DSM-IV criteria for gambling addiction. Participants responded to each question by selecting “yes,” “no,” “sometimes,” or “don't know.” The dependent variable was the number of questions to which they responded “yes” (1–13). The internal reliability for the current sample was acceptable (coefficient α = 0.60).

#### Barratt impulsiveness scale

The Barratt Impulsiveness Scale Version 11 (BIS-11; Patton et al., 1995) was used to measure general impulsivity. The BIS-11 is comprised of 30 statements (e.g., I change hobbies; I plan for job security) and for each statement participants selected among the following options: “Rarely/Never,” “Occasionally,” “Often,” or “Almost always/Always.” For scoring, responses were coded numerically from 1 (rarely/never) to 4 (almost always/always) and summed to obtain a total score (0–20). Higher scores indicate greater levels of impulsivity. The internal reliability of the BIS in the current sample was high (coefficient α = 0.75).

#### Risk-attitudes scale

A modified version of the Risk-attitudes Scale (RAS; Weber et al., 2002) included 20 statements from the ethical, gambling, and recreational subscales of the original measure. Participants indicated how likely or unlikely they would be to engage in the behavior described in each statement on a scale from 1 (very unlikely) to 5 (very likely). The dependent variable was the average score across all items (1–5). Higher scores reflect more accepting attitudes toward risk. The internal reliability of the measure in the current sample was high (coefficient α = 0.76).

#### Iowa gambling task

In the IGT (Bechara et al., 1994) participants selected one of four tokens on each trial in order to earn points. Each token was associated with its own set of gains and losses. Participants were instructed to try to earn as many points as possible before the end of the task. The gain or loss for each token was predetermined for each of the 100 trials, such that selecting two of the tokens (circle or square) on most trials results in a net gain of points, while selecting the other two tokens (crystal or diamond) on most trials results in a net loss of points. The participants were not told which tokens were “good” and which were “bad.” After a token was selected, the participant was informed of the outcome (gain or loss) and the total number of points they had earned. The tokens remained on the screen until the participant made a selection. The feedback was displayed for 1500 ms, and the response keys were “i” (circle), “r” (crystal), “c” (square), and “m” (diamond). The dependent variable was the number of times “bad” tokens were selected in the final 20 trials.

#### Temporal discounting

The TD task was similar to McClure et al. (2004). Participants stated their preference in a series of choices between a smaller amount of money received at an earlier time and a larger amount of money received at a later time. Participants were instructed to make each decision as if they would receive the option they selected. The first two choices were fixed to allow participants to learn how to respond in the task. The first choice required participants to select between the same amounts of money available at two different delays (e.g., $27.10 in 2 weeks vs. $27.10 in 1 month and 2 weeks) and the second choice required participants to select between two amounts of money in which the earlier amount is less than 1 percent of the later amount (e.g., $0.16 today vs. $34.04 in 1 month and 2 weeks). The remaining 40 trials were constructed by combining one of the early delays (today, 2 weeks, or 1 month) with one of the later delays (2 weeks, 1 month) and one of the following percent differences in amount of money: 1, 3, 5, 10, 15, 25, 35, 50%. The early amount of money was drawn randomly from a range of $5 to $40 and then the larger amount of money was set to the specified percent difference. All combinations of the early delays, late delays, and percent differences were used excluding those where the later delay would be more than 6 months after the experiment. The two options were displayed on either side of the screen with the smaller, earlier reward always presented on the left, and the options remained on the screen until a response was made. A yellow triangle located below each option turned red for 2000 ms after the response to indicate the selection. This was followed by a blank screen for 2000 ms and then the next choice appeared. Response keys were “v” for the option on the left and “m” for the option on the right. The dependent variable was the percentage of choices where the earlier/smaller amount of money was selected. Selecting the earlier option more frequently indicates greater risk aversion.

#### Probabilistic selection

In the probabilistic selection task (Frank et al., 2004), participants viewed three pairs of stimuli (AB, CD, EF) presented randomly and were instructed to select one of the stimuli in each pair. Probabilistic feedback was presented after each selection. In the first pair, selecting A led to positive feedback (i.e., “Correct!”) 80% of the time and selecting B led to negative feedback (i.e., “Incorrect”) 20% of the time. In the second pair, selecting C led to positive feedback 70% of the time, and in the third pair selecting E led to positive feedback 60% of the time. Participants performed three learning blocks of 60 trials (20 of each pair). In the final block, participants viewed all possible pairs of the six stimuli four times each and received no feedback about their choices. The stimuli were six Japanese Hiragana characters counterbalanced across the three feedback probabilities (i.e., AB, CD, EF). In all blocks, the figures remained on the screen until a response was made or until 4000 ms passed if no response was detected. In the learning blocks, feedback was displayed for 1500 ms. There was a 500 ms response-to-stimulus interval in the final block. Response keys were “v” to select the figure on the left and “m” to select the figure on the right. The dependent variables were the percentage of trials where A was chosen (Choose A) and B was avoided (Avoid B) in the final block. Greater selection of A than avoidance of B in the final block indicates learning based on positive rather than negative outcomes. Greater avoidance of B than selection of A in the final block indicates learning based on negative outcomes more than positive outcomes.

#### Risk task

In the risk task (Knoch et al., 2006), participants were presented with six boxes, each equally likely to contain a winning token. Some boxes were blue and others were pink. Participants were instructed to select the color of the box they believed to contain the winning token. If they chose correctly they received the number of points associated with the color they had selected, but if they were incorrect they lost that many points. Two variables were manipulated in this task. The level of risk refers to the ratio of pink and blue boxes which can be 5:1, 4:2, or 3:3. For example, if there are 5 blue boxes and 1 pink box, then there is a 1 in 6 chance that the pink box contains the winning token; therefore selecting pink would be riskier than selecting blue. The balance of reward refers to the number of points the colors are worth and can be 90:10, 80:20, 70:30, or 60:40. The color with fewer boxes was always worth the greater point value. In the example above, for instance, selecting pink would be worth 90 points while selecting blue would only be worth 10 points. Participants completed 100 trials. Four of these were combinations of the 3:3 level of risk with balance of reward and were not included in the analysis. The remaining 96 trials included all other possible combinations of level of risk, balance of reward, and color. The level of risk was displayed above the boxes on each trial and the balance of reward was displayed below. The box displays remained on the screen until the participant responded followed by feedback displaying the outcome and total points for 1500 ms. The response keys were “v” to select pink and “m” to select blue. The dependent variables for this measure were the total score at the end of the task (Risk Total) and the percentage of low risk selections (Low Risk).

### Procedure

All stimuli were presented using E-Prime 1.2 Software (Psychology Software Tools, Pittsburgh, PA). Participants signed the informed consent and completed the BIS-11, pathological gaming scale, RAS, and the media usage questionnaire. Half of the participants completed the tasks in the following order: TD, risk task, Iowa Gambling Task, and Probabilistic Selection; the other half of the participants completed the tasks in the reverse order. Participants also completed the useful-field of view and stop-signal tasks, but as these data do not specifically address the relationship between video games and risky decision making, it is not reported here. After the tasks were completed the participants were debriefed and thanked for their participation. The entire study took ~90 min.

## Results

### Sample characteristics

Table 1 includes the means, standard deviations, and ranges of all the measured variables. More than half of the sample (64%) reported playing video games at least 2 h per week. The average amount of time reported playing video games was 20.6 h per week (*SD* = 25.4, 25th quartile = 0, 50th quartile = 13, 75th quartile = 34). Males reported playing more hours per week (*M* = 28.2, *SD* = 21.9) than females (*M* = 12.1, *SD* = 26.5), *t*_(147)_ = 4.06, *p* < 0.001. Pathological gaming (i.e., responding “yes” to 6 or more of the statements on the PVP scale) was reported by 7.4% (males = 13.9%, females = 0%) of the sample, consistent with the rate observed in other samples of children and adolescents (Gentile, 2009; Gentile et al., 2011). The mean number of pathological gaming symptoms was, *M* = 1.8, *SD* = 2.0. Males reported more symptoms related to pathological gaming (*M* = 2.7, *SD* = 2.1) than females (*M* = 0.8, *SD* = 1.2), *t*_(147)_ = 6.90, *p* < 0.001.

**Table 1:** **Descriptive statistics for all independent and dependent variables**.

	***M***	***SD***	**Range**
Hours	20.63	25.38	0–139
FPS	–	–	0–1
Strategy	–	–	0–1
PVP	1.84	1.98	0–8
BIS-11	65.48	8.93	46–90
RAS	2.17	0.55	1.05–3.75
IGT	10.91	4.32	0–20
TD	0.73	0.21	0–1.0
Choose A	0.63	0.21	0–1.0
Avoid B	0.65	0.24	0.06–1.0
Low risk	0.83	0.14	0.30–1.0
Risk total	318.52	698.51	−2360 to 1240

### Zero-order correlations

Correlations among all of the variables included in the analyses are presented in Table 2. The pattern of association observed in these variables is briefly summarized before consideration of the results of the CCA in order to orient the reader to the fundamental relationships that are present in the dataset. In addition to the observed variables, five two-way interaction terms were computed (i.e., the number of hours spent playing video games per week (hours) with PVP and the two genres of video games (i.e., FPS and strategy), and PVP with the two genres). Sex (dummy coded: Male = 1, Female = 2) was negatively correlated with Hours, FPS, PVP, RAS, Hours × PVP, Hours × FPS, Hours × Strategy, PVP × FPS, and PVP × Strategy, indicating that males reported greater video game experience, pathological gaming and risk-taking than females. Hours was positively correlated with PVP, PVP × FPS, and PVP × Strategy. FPS gaming was positively correlated with PVP. Strategy gaming was positively correlated with PVP and Hours × PVP. The number of pathological gaming symptoms was positively correlated with Hours × FPS and Hours × Strategy. These data indicate that the prevalence of pathological gaming increases with the number of hours spent gaming per week, and that this is true for both FPS and strategy games.

**Table 2:**
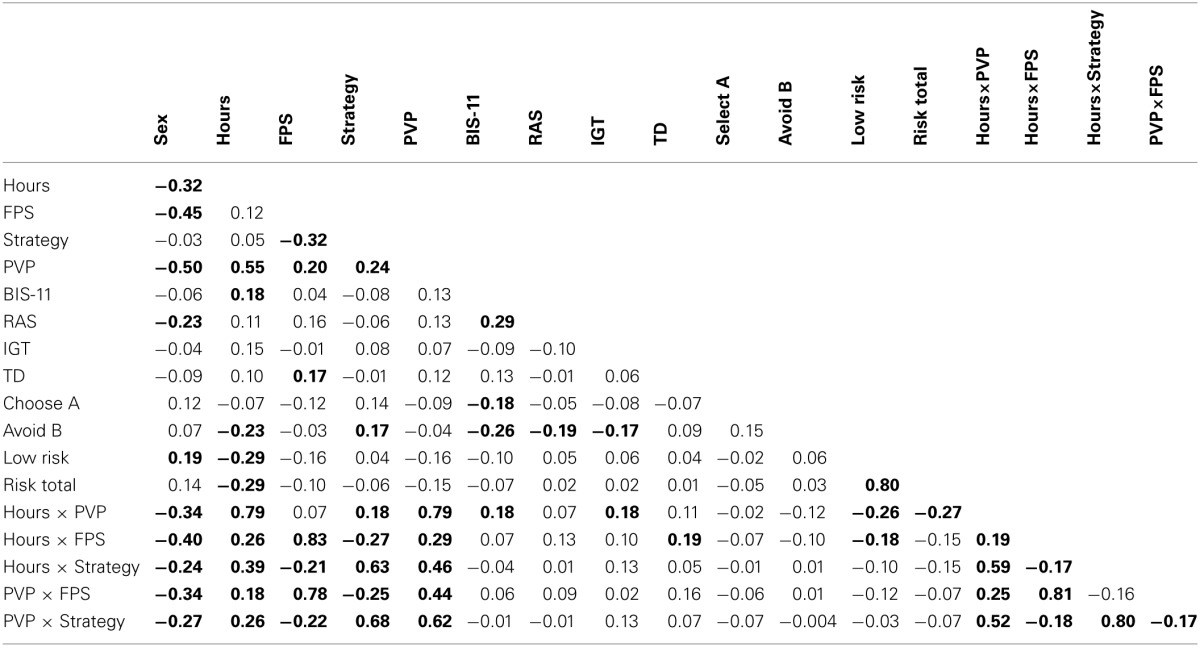
**Correlations between all variables and interaction terms**.

***p*** < ***0.05***.

**Figure 1:**
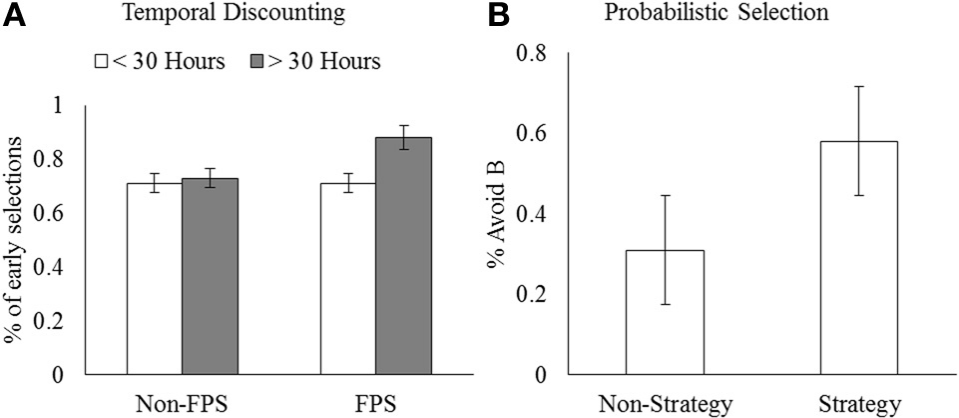
**(A)** Mean proportion of early selections in the temporal discounting task as a function of Hours and identification as an FPS gamer. **(B)** Avoid B in the probabilistic selection task as a function of identification as a strategy gamer. Error bars represent standard error of the mean.

Consistent with our hypotheses, there were two patterns of association between video game experience and the measures of risky decision making (i.e., increased impulsivity, reduced sensitivity to negative feedback). Self-reported impulsivity was positively correlated with Hours and Hours × PVP, consistent with previous work (Gentile et al., 2011). Selection of the earlier, smaller reward in the TD task was positively correlated with FPS [FPS gamers: *M* = 0.79, *SD* = 0.17; non-FPS gamers: *M* = 0.71, *SD* = 0.22; *t*_(147)_ = −2.10, *p* = 0.04] and Hours × FPS (Figure 1A), consistent with the hypothesis that this genre of video game can shift an individual's focus toward immediate rewards, resulting in more impulsive decision making.

**Figure 2:**
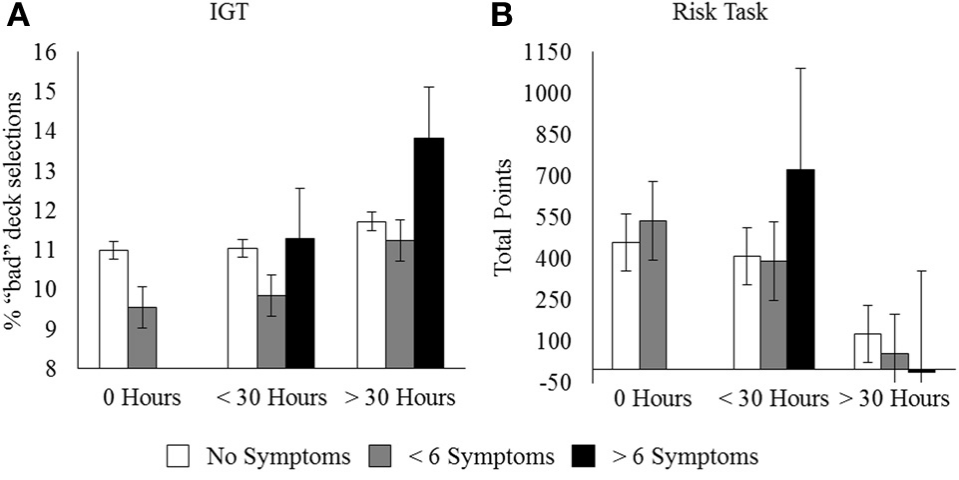
**(A)** Proportion of trials on which the “bad” decks were selected in the IGT and **(B)** total points earned in the risk task as a function of Hours and PVP symptoms. Error bars represent standard error of the mean.

Selection from bad decks in the IGT was positively correlated with Hours × PVP (Figure 2A), supporting the idea that increased hours and pathology are related to reduced learning from negative outcomes. The percentage of low risk selections in the risk task was negatively correlated with Hours, Hours × PVP, and Hours × FPS, indicating greater risk taking among gamers. Importantly, the total score in the risk task was negatively correlated with Hours and Hours × PVP (Figure 2B), demonstrating that selecting the riskier option more frequently had a detrimental effect on overall gains for individuals with more gaming experience and PVP symptoms. Similarly, sensitivity to negative feedback in the probabilistic selection task was negatively correlated with Hours, further indicating failure to learn from negative outcomes. In contrast, sensitivity to negative feedback was positively correlated with Strategy games (Figure 1B). Strategy gamers (*M* = 0.72, *SD* = 0.25) avoided B more frequently than non-strategy gamers (*M* = 0.62, *SD* = 0.23), *t*_(146)_ = −2.09, *p* = 0.04, supporting the hypothesis that this genre may encourage players to learn from mistakes and avoid making them in the future.

### Canonical correlation analysis

**Figure 3:**
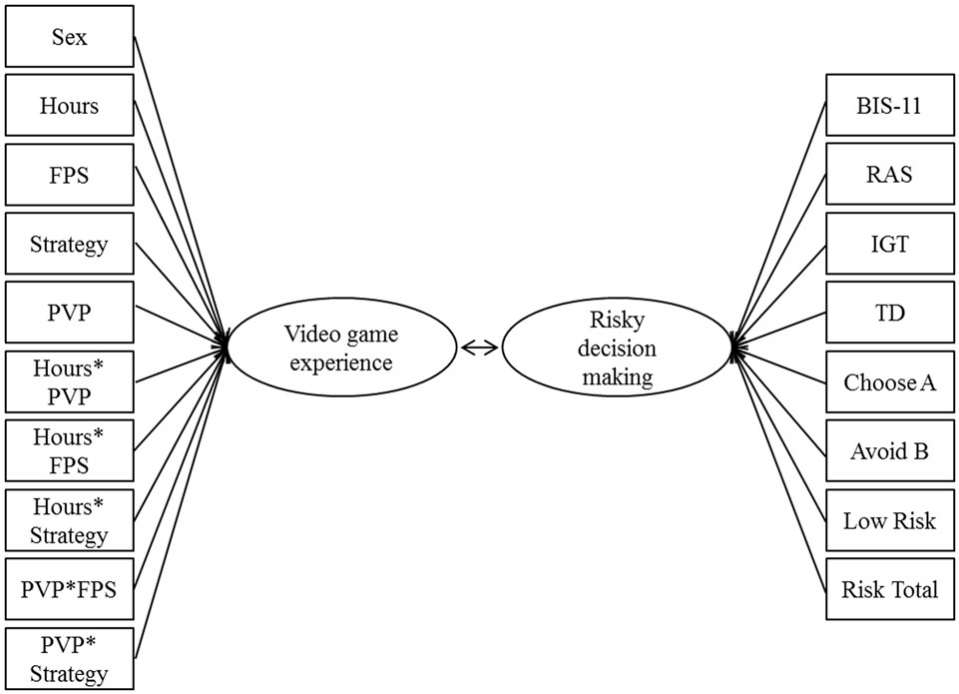
**Illustration of the canonical correlation function with ten predictors (boxes on the left side) and eight dependent variables (boxes on the right side).** The canonical correlation is the Pearson's *r* between the two latent variables (ovals), which are derived from the observed variables.

To examine the latent associations between video game experience and pathology (i.e., predictor variables), and risky decision making (i.e., dependent variables; Figure 3) a CCA conducted. The advantages of using this approach and its assumptions have been outlined in Sherry and Henson (2005). Importantly, CCA reduces the chance of Type I error (i.e., spurious significant associations) while allowing an investigator to evaluate the multivariate shared relationships between the two sets of variables (i.e., video game experience and risky decision making). The analysis revealed nine functions with squared canonical correlations (*R*^2^_c_) of 0.39, 0.28, 0.19, 0.14, 0.11, 0.05, 0.03, 0.02, and 0.01 for functions one through nine, respectively. The full model was significant using the Wilks's λ = 0.25 criterion, *F*_(117, 955)_ = 1.68, *p* < 0.001. Wilks's λ represents the variance unexplained by the model, therefore 1—Wilks's λ represents the full model effect size in terms of *r*^2^. In this analysis with nine canonical functions, the *r*^2^ was 0.75, indicating that the full model explained 75% of the variance between the two sets of variables. To test the hierarchical arrangement of the functions for statistical significance, a dimension reduction analysis was used (Table 3). The test of the full model was significant (i.e., Functions 1–9), as was the test of Functions 2–9. Together these two functions explained 67% of the variance. None of the other functions explained a significant proportion of the shared variance between the variable sets after extraction of the prior functions. The first canonical function revealed a correlation of *r* = 0.62 between the predictor and dependent variables, and the second canonical function revealed a correlation of *r* = 0.53 between the variable sets. This indicates that for the first two canonical functions the two variable sets were highly correlated (Sherry and Henson, 2005).

**Table 3:** **Tests of canonical functions**.

**Function**	**Wilks λ**	**Canonical correlation**	***F***	***df*1**	***df*2**	***p***
1–9	0.24	0.62	1.68	117	955	0.001
2–9	0.40	0.53	1.3	96	866	0.03
3–9	0.56	0.44	1.02	77	775	0.44
4–9	0.69	0.37	0.82	60	681	0.83
5–9	0.80	0.33	0.65	45	585	0.96
6–9	0.90	0.23	0.44	32	485	0.99
7–9	0.94	0.17	0.34	21	380	0.99
8–9	0.98	0.13	0.26	12	266	0.99
9–9	0.99	0.07	0.14	5	134	0.98

**Figure 4:**
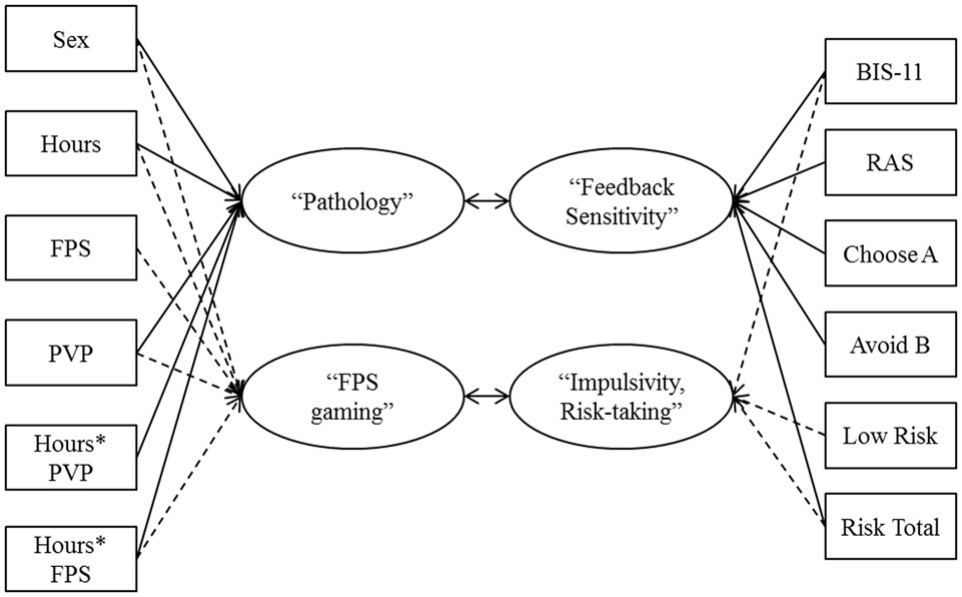
**Graphical representation of the significant canonical functions and the strongest contributing predictors (left side) and dependent variables (right side).** Solid lines represent the first canonical function and dashed lines represent the second canonical function.

The canonical correlations between the variables (predictor and dependent) and the functions indicate which variables have the strongest contribution to the function and can be interpreted in a similar manner as the factor loadings in a factor analysis (Afifi et al., 2004). In a sample of 148, an *r* of 0.30 is significant at the 0.001 level; therefore variables for which *r* ≥ 0.30 were considered statistically significant (Table 4). Consistent with our predictions, the first function represents a positive association between hours and pathological gaming, and risk-taking, impulsivity, and differential learning from positive and negative feedback (Figure 4). Specifically, the first canonical function explained 11.12% of the variance in the dependent variables and was most strongly related to RAS, Risk Total, Avoid B, Choose A, and BIS-11. With the exception of RAS, the sign of the correlation was the same for all variables, indicating that they were positively related. RAS scores were inversely related to the other variables, meaning that higher scores on the RAS were associated with lower total scores on the risk task. The first function explained 5.34% of the variance in the predictor variables with primary contributions from Hours × PVP, Hours, Sex, PVP, and Hours × FPS. All of these variables, except sex, were positively related to the dependent variables, indicating more hours, pathological symptoms, and times spent playing FPS games predicted impulsivity, sensitivity to feedback, and losses on the risk task. The negative association with sex indicates that males engaged in more risky decision making than females.

**Table 4:** **Canonical correlations after varimax rotation of the dependent variables**.

	**Canonical function**	
	**1**	**2**
**DEPENDENT VARIABLES**		
BIS-11	**0.30**	**0.74**
RAS	**−0.84**	−0.23
IGT	0.27	0.26
TD	0.02	−0.14
Choose A	**0.32**	0.17
Avoid B	**0.34**	0.10
Low risk	0.19	**−0.32**
Risk total	**0.38**	**0.59**
**VIDEO GAME MEASURES AND SEX**		
Sex (1 = male, 2 = females)	**−0.52**	**−0.64**
Hours	**0.62**	**0.33**
FPS	0.19	**0.38**
Strategy	0.07	−0.23
PVP	**0.52**	**0.34**
Hours × PVP	**0.67**	**0**.21
Hours × FPS	**0.35**	**0.36**
Hours × Strategy	0.27	0.20
PVP × FPS	0.24	0.27
PVP × Strategy	0.23	0.23

***p*** < ***0.001***.

The second function explained 8.08% of the variance in the dependent variables and was most strongly related to BIS-11, Risk Total, and low risk selections. As expected, making low risk selections was associated with higher scores on the risk task and lower impulsivity. The second function explained 2.94% of the variance in the predictor variables and was primarily related to FPS, Hours × FPS, PVP, Hours, and sex. Consistent with our hypotheses, experience with FPS video games and PVP symptoms predicted worse performance on the risk task (e.g., fewer low risk selections and lower total scores) and greater impulsivity (Figure 4). In contrast to the first function, BIS-11 scores were more strongly predicted here, emphasizing the effects on impulsivity and supporting previous work (Gentile, 2009; Gentile et al., 2011).

## Discussion

The current study was designed to examine the relationships between video game experience, pathological gaming, and risky decision making. Consistent with previous work (Gentile, 2009; Gentile et al., 2011), ~7% of the current sample of young adults met criteria for pathological gaming. Moreover, pathological gaming was not observed in females in our sample. Given the gender balance in the sample, this means that roughly 14% of those males that participated in the study reported pathological gaming. Significant correlations were observed between hours spent gaming, pathological gaming and game genre, and impulsivity, risk-taking and sensitivity to positive and negative feedback. The CCA revealed that pathological gaming was positively related to feedback sensitivity, while playing FPS games was positively related to impulsivity and risk-taking.

The self-report and behavioral measures revealed that pathological gaming and playing FPS games were positively associated with greater impulsivity. The interaction between hours and PVP was also positively related to BIS-II scores indicating that more pathological symptoms were positively associated with greater impulsivity (Gentile et al., 2011). Complementing this finding, the second canonical correlation represented the association between pathological gaming, FPS gaming, and impulsivity. Evidence for the TD task also support the idea that game genre may influence the association between gaming and impulsivity. For the TD task, selection of the smaller reward that is delivered earlier could be taken as an index of impulsivity (Mitchell, 1999; Ohmura et al., 2005). In this task, selection of smaller rewards was positively associated with the playing FPS video games, but not with playing strategy video games. The association between FPS gaming and impulsivity is interesting, given evidence that this form is gaming is also associated with a reduction in the use of proactive cognitive control (Bailey, 2009; Bailey et al., 2010; Swing, 2012). Together these data may indicate that playing FPS games and pathological gaming are associated with an increase in impulsive behavior that results from a decrease in the utilization of proactive cognitive control to guide behavior.

The association between gaming and risky decisions was sensitive to game genre. In the risk task, the number of hours spent playing video games, the interaction between hours and PVP, and categorization as an FPS gamer were all negatively correlated with the percentage of low risk selections; and hours predicted selecting the high-risk option more frequently in the CCA. This was accompanied by a dramatic reduction in the total points earned at the end of the task, indicating that the selection of risky options in the risk task did not pay off in the end. Taken together, these findings provide clear evidence that gaming time, pathology, and FPS games influence an individual's selection of risky options, and this behavior appears to continue in spite of its detrimental effect on performance over time. In contrast to FPS gaming, strategy gaming was not as strongly related to increased risk-taking. One explanation for the differential influence of game genre is that there are likely to be social repercussions for making impulsive decisions in a strategy game since successful in the game often requires cooperation with a team. It is important to note that both strategy games and FPS games were positively correlated with PVP and the correlation between pathological symptoms and hours × strategy (*r* = 0.46) appeared to be higher than the correlation between PVP and hours × FPS (*r* = 0.29), although this difference did not reach significance, *t*_(146)_ = 1.53, *p* > 0.05. This suggests that both strategy and FPS games are associated with pathological gaming, but that the consequences for impulsivity and risk-taking are not the same for the two genres. This may be due to the structure of the gaming environment or the goals of the players within the different genres.

Performance on the risk task, the probabilistic selection task, and to a lesser degree the IGT provides some evidence that gaming and pathology are positively associated with decreased sensitivity to negative outcomes. Game time was positively correlated with poorer performance on the risk task (e.g., lower total score) due to greater selection of risky options. Presumably, after several selections of the low chance risky options, the accruement of losses should be a deterrent for further selection of the risky option, but this did not appear to be the case. Similarly, feedback over several trials of the IGT should result in decreased selection from the “bad” decks. Higher PVP scores and hours gaming were associated with greater selection from the “bad” decks well past the point at which the feedback was effective at decreasing selection from these decks among non-pathological high gamers.

The probabilistic selection task (Frank et al., 2004) provided insight into whether or not reinforcement learning driven by positive or negative feedback was sensitive to gaming. Increased FPS gaming and gaming pathology was associated with a decrease in avoiding B (i.e., learning from negative feedback). However, strategy games were positively correlated with avoidance of B (i.e., *r* = 0.17), suggesting that individuals who identify as strategy gamers are more sensitive to negative feedback. As with impulsivity, the characteristics of strategy games may explain this relationship. Mistakes in a strategy game can have long-term consequences for achieving goals in the game because game play usually spans a longer time frame than an FPS video game. Therefore, mistakes in a strategy video game can be costly, and one would benefit from paying attention to negative outcomes and learning to avoid those outcomes in the future.

There are a few limitations of the current study worth noting. First, the design was not experimental and this has two implications. It is possible that some unmeasured variable(s) account for the findings, and the direction of causation cannot be defined (i.e., does playing games increase risk taking and impulsivity or are impulsive individuals drawn to video games?). Future studies may address this issue by looking at the short- and long-term effects of video game training on risky decision making, similar to work on aggression (Anderson et al., 2010) and visuospatial processing (Bavelier et al., 2012). Second, only two genres of video games were examined, however, the genres examined in the study tend to be the most popular among gamers (The NDP Group, 2010). Based on the current data and other work (e.g., Fischer et al., 2009), it appears that the association between are video game experience and risky decision making is likely to be moderated by the genre of video game, with some effects being specific to one particular genre (Green and Bavelier, 2003). Further research will be necessary to better understand the effects of different genres and how these effects may interact in individuals who play more than one genre. Finally, the current study focused primarily on risky decision making in the context of gambling (i.e., participants were attempting to gain points in the decision making tasks), therefore the data do not speak to risk-taking in other contexts, such as social or academic behavior. Other studies have demonstrated the effects of driving video games on attitudes toward and engagement in risky driving behavior (Beullens et al., 2011), so together with the current findings it appears that video games may influence risk-taking in closely related contexts as well as in more dissimilar contexts (e.g., FPS games predict performance in the risk task).

The current study extends the literature on the relationship between video game experience and risky decision making beyond risky driving behavior (Fischer et al., 2009; Beullens et al., 2011), and indicates that pathological symptoms and genre play a pivotal role in determining the relationship between gaming experience and decision making. We have demonstrated that pathological video game use is associated with increased impulsivity, greater risk-taking, and greater losses in gambling-like tasks. These laboratory based findings are consistent with real-world reports of the consequences of excessive video game playing, including family discord (Warren, 2011), financial loss (Doan and Strickland, 2012), and even death (BBC News, 2005). In the current sample, both FPS and strategy video games, two popular genres, were positively correlated with pathological symptoms. This work underlines the importance of further research to understand the etiology and treatment of pathological video game use, and to explore the effects of different genres.

### Conflict of interest statement

The authors declare that the research was conducted in the absence of any commercial or financial relationships that could be construed as a potential conflict of interest.
